# Management of Crown Root Fracture by Interdisciplinary Approach

**DOI:** 10.1155/2013/138659

**Published:** 2013-09-26

**Authors:** K. Radhakrishnan Nair, Anoop N. Das, Manoj C. Kuriakose, Nandakumar Krishnankutty

**Affiliations:** ^1^Department of Conservative Dentistry and Endodontics, Azeezia College of Dental Sciences and Research, Kollam 691537, India; ^2^Department of Periodontics, Azeezia College of Dental Sciences and Research, Kollam 691537, India

## Abstract

Fracture of tooth after trauma is distressing to a person because of the discomfort and pain due to pulpal injury. Crown root fractures of anterior teeth cause concomitant periodontal injury and there will be concern about appearance, and aesthetics. Management of pulpal and periodontal tissue relieves pain and restoration of tooth form regains patients confidence. Restoration of fractured tooth will be accepted readily if it is minimally invasive, less expensive, and aesthetically acceptable. Reattachment is an option for restoration of anterior teeth compared to other artificial replacements because of its appearance as natural. This method is favourable when the fractured fragment is intact and available. Utilization of pulp space for retention of fragment is achieved by the insertion of a dentine bonding post. This case report describes a case of tooth reattachment after trauma in which the pulp space is utilized to bond a fiber-reinforced post for retention after periodontal tissue management.

## 1. Introduction

Tooth fracture can occur at any age due to trauma. Sports accidents and fights are more common among teenagers and automobile accidents are seen in all age groups. Impact of trauma on tooth varies from mild enamel chipping to complex crown root fractures. Aesthetic and functional implications of tooth fracture depend upon its severity and age of the patient. About 5% of all dental traumas are found to be associated with crown root fractures [1]. Severe pain arising from crown root fractures can be either due to pulpal exposure or due to concomitant periodontal injury or both.

Clinical considerations for the management of crown root fractures include extent and pattern of fracture, restorability of remaining tooth, availability of fractured fragment, and damage to the attachment apparatus [2, 3]. Extension of fracture subgingivally raises concern about biological width violation. Periodontal flap surgery combined with osteoplasty procedures is indicated for deep subgingival fractures to satisfy the requirement of biological width [4].

A conservative method for management of the crown root fracture when the intact fragment is available is the reattachment technique. It is a method which has been tried long before [5]. This method is gaining wide acceptance because of its several advantages over artificial replacements like composite resin or full coverage restorations. It can offer long lasting aesthetics and is reasonably a simple procedure [6, 7].

It is cost effective and can be completed in less chair side time. When endodontic therapy is indicated, the pulp space available after obturation can be used for retention of the fragment by using posts bonded to root canal. This case report describes an interdisciplinary approach to the management of two complicated crown root fractures of maxillary central incisors after an automobile accident.

## 2. Case Report

A forty-year-old male patient was referred to the Department of Conservative Dentistry and Endodontics with the complaint of broken front teeth. He had a history of road traffic accident with sustained hand and facial injuries and had fracture of two maxillary central incisors. He went to a nearby hospital immediately because of pain for medical aid and took some medications. He had no relevant medical history and reported to the department next day due to elevation of pain in the area.

**Figure 1:**
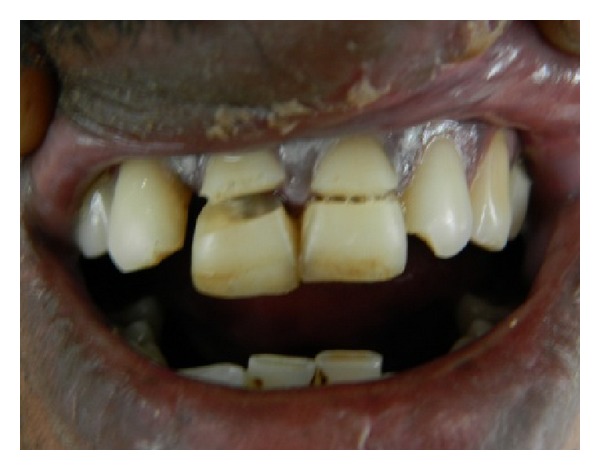
Preoperative view.

Extra oral examination revealed lacerations with swelling of upper lips. Intraorally lacerations were present on buccal mucosa. Gingiva appeared to be erythematous in the upper front region and there was bleeding on probing. Both the upper central incisors were fractured with pulpal exposure. The horizontal fracture line was on the middle of the labial surface extending obliquely to the subgingival area on the palatal side (Figure 1). The incisal fragments were mobile and during talking, pain increased due to mobility. An IOPA radiograph showed the crown of 11 and 21 with fracture line extending subgingivally and the root and periapical area was found to be normal. This was diagnosed as a case of complicated crown root fracture with irreversible pulpitis.

**Figure 2:**
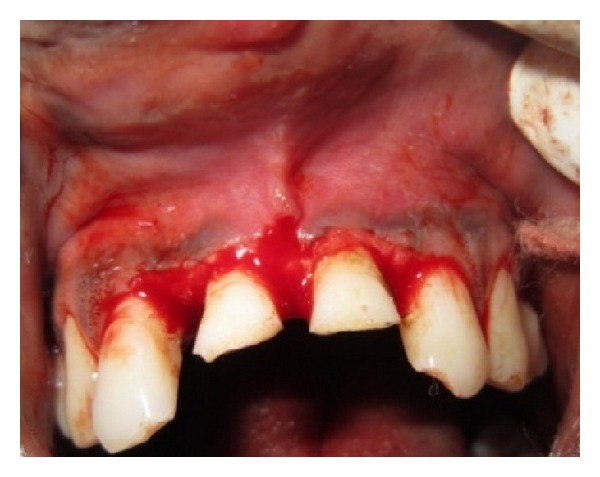
Gingivectomy of 11 and 21.

**Figure 3:**
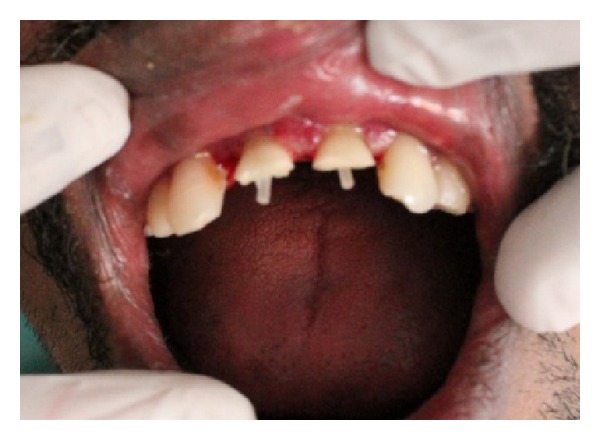
After fiber post fixation.

 The incisal fragments were removed after local anesthesia as a single piece and kept in normal saline immediately. The various options to restore the teeth were explained to the patient. After listening, the patient expressed the willingness to reattach the broken part. Single visit endodontics was done and root canal was obturated with gutta-percha using AH Plus as the sealer. Gingivectomy was done for 11 and 21 to bring the fracture line supragingival (Figure 2). The patient reported to the clinic after one day for the reattachment procedure. Root canal preparation for the post was done sequentially using the Tenax Fiber Trans drill (Coltene Whaledent). Corresponding fiber reinforced composite post was selected (Tenax Fiber Trans-Coltene Whaledent) to check the fit and occlusal clearance. The occlusal end of the post was shortened with a diamond disc to the desired length. The prepared root canal was conditioned using self-etching non rinse conditioner (ParaBond-Coltene Whaledent). Fixing of the post in the root canal was done using dual cure luting material (ParaCore-Coltene Whaledent). To facilitate polymerization, curing LED light (Woodpecker) was applied through the tip of the post into the root canal for 20 seconds. About 2 mm of the post was visible beyond the incisal margin after fixing (Figure 3).

**Figure 4:**
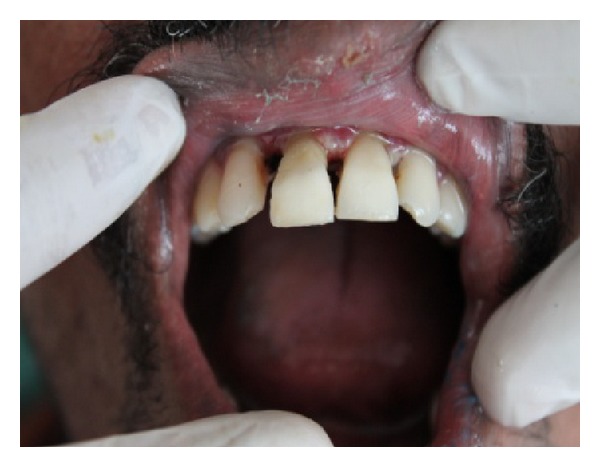
Immediate postoperative view.

A small recess was prepared in the pulp chamber of fractured segment of 11 and 21 and was tried against the remaining crown portion with the post for approximation. The opposite surface of the fractured crown was then etched with 37% phosphoric acid and the fragment was luted in the correct position using dual cure resin (ParaCore-Coltene Whaledent) with slight pressure. Excess of the material was removed using a sharp instrument from the edges and was light cured for 20 seconds for faster polymerization. A bevel was prepared on the margins of the approximating surfaces of 11 and 21 on the labial side and the margins were sealed with nanocomposite (Brilliant NG-Coltene Whaledent). Polishing of the surface was done with polishing disks which ensured an aesthetic blending of the margins (Figure 4).

**Figure 5:**
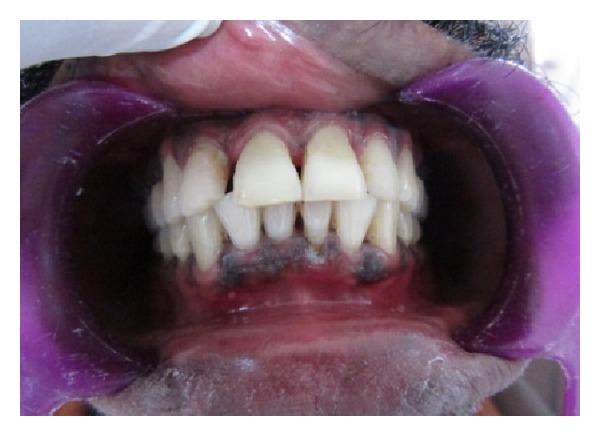
Postoperative view after one year.

**Figure 6:**
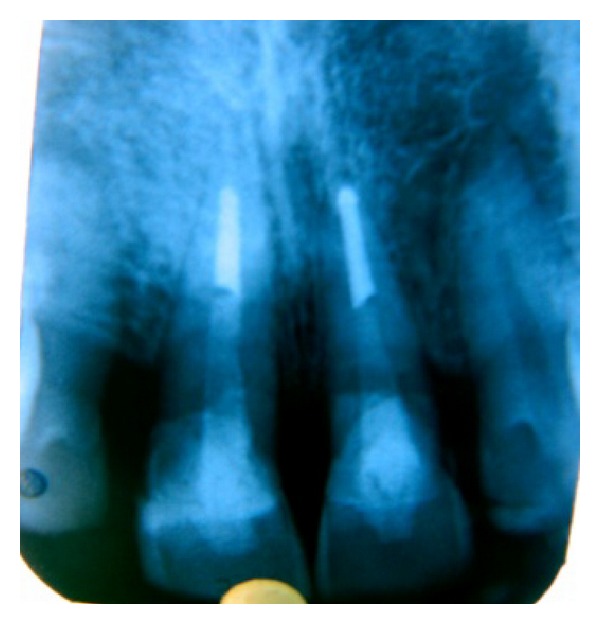
Postoperative radiograph after one year.

Patient was recalled after six months and one year. On examination, 11 and 21 were found to be asymptomatic with satisfactory aesthetics. Periodontal status was good with 1 mm pocket. Gingival tissues had a normal texture with a normal contouring (Figure 5). Intraoral periapical radiograph showed intact tooth structure with intact lamina dura (Figure 6).

## 3. Discussion

Fracture of anterior teeth after trauma adversely affects the emotional well-being of a person in addition to the discomfort and pain. Complexity and extension of fracture along with the associated injury to the tooth influence the restorative design. Reattachment of the tooth is an option when the broken fragment is intact and available. It has several advantages over conventional methods of restoration. It retains the translucency of natural tooth and its abrasive resistance is better than composites.

It is less time consuming and is cost effective. Several studies have shown that the impact strength of reattached tooth is not significantly different from that of intact natural tooth [8, 9]. 

Reattachment procedure is often multidisciplinary dictated by the extension of tooth fracture and injury to the attachment apparatus. This case was a subgingival fracture and gingivectomy was done to bring the fracture line supragingival. Pulp space after root canal treatment was utilized to attach a post for auxiliary retention. Metallic and nonmetallic posts are available with different properties. Fiber posts which have the modulus of elasticity similar to that of root dentin are used here to bond with the root and are preferred to metal posts because of less stress concentration on the root and there is low incidence of root fracture [10]. There is less tooth preparation with a fiber post compared to cast post; thus, the tooth is conserved more. Failures of post and core occur by debonding of the core and due to root fracture [11]. 

Available clinical evaluation for longevity of reattachment shows medium-term prospects for this technique [12, 13]. A seven-year follow-up of crown reattachment showed mild discoloration of crown without any evidence of fracture [14]. Long-term followup is required to assess the longitivity of reattachment technique. Improvement in adhesive technology may provide a long-lasting bonding of the fragments to improve the prospects of this technique in future.
